# An ns-3 Evaluation Framework for Receiver-Initiated MAC Protocols with Configurable Enhancement Modules Across Various Network Scenarios

**DOI:** 10.3390/s26010164

**Published:** 2025-12-26

**Authors:** Tomoya Murata, Shinji Sakamoto, Takashi Kawanami

**Affiliations:** 1 Information and Computer Engineering Program, Graduate School of Engineering, Kanazawa Institute of Technology, 7-1 Ohgigaoka, Nonoichi 921-8501, Japan; c1039548@planet.kanazawa-it.ac.jp; 2 Department of Artificial Intelligence, College of Information Science and Engineering, Kanazawa Institute of Technology, 7-1 Ohgigaoka, Nonoichi 921-8501, Japan; 3 Department of Information and Computer Science, College of Information Science and Engineering, Kanazawa Institute of Technology, 7-1 Ohgigaoka, Nonoichi 921-8501, Japan; t-kawanami@neptune.kanazawa-it.ac.jp

**Keywords:** receiever-initiated, ns-3, WSN, IoT

## Abstract

Receiver-initiated MAC protocols, such as the IEEE 802.15.4e RIT scheme, are promising for energy-efficient communication in multi-hop wireless sensor networks. However, their practical use requires a better understanding of how multiple contention-avoidance mechanisms interact under realistic network conditions. This study develops an ns-3 implementation of an RIT-compliant receiver-initiated MAC protocol together with a flexible evaluation framework that enables selective activation of representative enhancement strategies, including carrier-sensing options for data and beacon transmissions and randomization of beacon intervals. Four realistic network scenarios were designed to simulate practical deployment settings. Simulation results revealed that the effectiveness of these enhancement strategies varied significantly depending on network load and topology. In particular, beacon interval randomization, although often assumed to improve robustness, was found to degrade performance under low-load conditions, indicating that even widely adopted mechanisms may behave differently depending on operational environments. Conversely, CSMA-based approaches provided consistent improvements in transmission reliability. These observations highlight the importance of considering environmental factors and parameter configurations when enabling enhancement mechanisms. Overall, the proposed platform provides a reproducible and unified environment for fair comparison of receiver-initiated MAC protocols and their optional mechanisms, offering practical insights for selecting appropriate configurations in real sensor network deployments.

## 1. Introduction

Receiver-initiated MAC protocols have been studied as a practical approach for realizing asynchronous and energy-efficient wireless sensor networks. Because their operation does not rely on global time synchronization, they avoid the communication and computational overhead associated with maintaining clock alignment. Moreover, they do not require high-precision oscillators, allowing the use of inexpensive sensor nodes with low clock accuracy. This makes receiver-initiated communication suitable for systems with strict constraints on device cost and energy consumption.

Compared with other asynchronous low-power MAC protocols, receiver-initiated communication provides better efficiency in channel usage. For example, CSL (Coordinated Sampled Listening) operates by having the receiver periodically sample the channel, while the sender repeatedly transmits wake-up frames until the receiver becomes active. This mechanism results in many control frames being sent before communication is established, which increases the bandwidth consumption. In contrast, receiver-initiated protocols use a single beacon sent periodically by the receiver as the trigger for data transmission. Since the sender transmits data frame after detecting the beacon, the overall channel usage can be reduced, which contributes to higher efficiency, especially in dense networks.

Despite these advantages, receiver-initiated MAC protocols also exhibit several structural challenges that arise from their beacon-driven operation. When multiple transmitters react to the same beacon, sender-side contention can easily occur, and this issue becomes more pronounced in dense deployments or under increased traffic load. The beacon interval further introduces a fundamental trade-off between energy consumption and delay: shorter intervals reduce latency but increase duty cycle, whereas longer intervals improve energy efficiency at the cost of throughput. These limitations are particularly evident in multi-hop networks, where forwarding nodes must wait for beacons from upper-layer nodes before transmitting. As waiting times accumulate toward the sink, traffic can become concentrated near the root of the network, leading to congestion and degraded performance. Nevertheless, many existing studies evaluate receiver-initiated protocols only under simplified or idealized conditions, such as single-hop communication or uniform traffic patterns, which do not fully capture the interactions between MAC-layer behavior, topology, and network load. A more comprehensive evaluation therefore requires an environment capable of examining protocol behavior across diverse network structures and traffic conditions.

Another difficulty in studying receiver-initiated MAC protocols is that many papers do not describe the implementation details in a clear manner. Important behaviors, such as switching between sender and receiver states, handling timeouts after transmission failures, or defining the exact frame structure, are often left ambiguous. Although receiver-initiated MACs are conceptually simple, these implementation decisions directly affect communication success rate and energy consumption, and differences in implementation can lead to inconsistent behavior across studies. As a result, it is difficult for researchers to reproduce the same operating conditions, which reduces the reproducibility of protocol comparisons and evaluations.

In this study, we implement the receiver-initiated transmission (RIT) defined in IEEE 802.15.4e [1] on the ns-3 [2] simulator. The implementation clarifies essential behaviors of the protocol, including state transitions between sending and receiving, timeout handling, and the conditions under which a node shifts into the sender role. We also incorporate two mechanisms proposed in previous work—simple carrier sensing (Pre-CS) and randomized beacon timing—as optional modules. Using this implementation, we construct an evaluation environment that supports end-to-end analysis from the MAC layer to the application layer under unified conditions.

The aim of this work is not to determine the superiority of specific design options, but to provide a reproducible and extensible basis for comparing different mechanisms of receiver-initiated MAC protocols. The extended ns-3 code and evaluation scripts will be made publicly available after the publication of this paper.

### Contributions

The main contributions of this study are summarized as follows:

- RIT-compliant MAC implementation in ns-3: A new implementation of an IEEE 802.15.4e RIT-based receiver-initiated MAC was developed for ns-3, clearly defining its core behaviors such as state transitions, sender/receiver switching, and timeout handling. The implementation is publicly available to support reproducibility and further research (the source code is available at: https://github.com/kawalab/ns3-rit-mac accessed on 19 December 2025).
- Modular integration of representative enhancement mechanisms: Key enhancement mechanisms, including simplified carrier sensing and beacon randomization, were modularized and incorporated into a unified multi-layer evaluation environment.
- Identification of environment-dependent behavior: Simulation results revealed that the effectiveness of enhancement mechanisms is highly dependent on network conditions, and that beacon randomization may degrade performance under low-load settings.

These contributions collectively provide a reproducible and comparable evaluation platform for receiver-initiated MAC protocols, supporting fair comparisons and future improvements in subsequent studies.

## 2. Background and Related Work

### 2.1. Overview of Receiver-Initiated MAC Protocols

**Figure 1:**
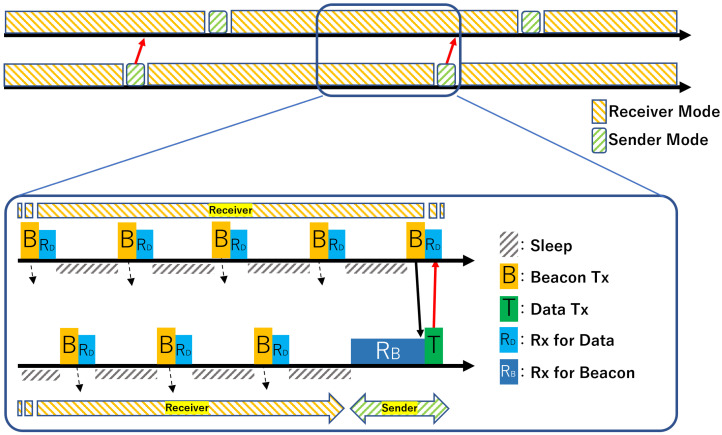
Basic operation of IEEE 802.15.4e RIT.

Receiver-initiated MAC protocols are characterized by the fact that, unlike traditional sender-initiated MAC protocols such as B-MAC [3] and X-MAC [4], the receiver initiates the opportunity for communication. The basic operation of the IEEE 802.15.4e RIT procedure is illustrated in Figure 1.

After waking up, the receiver node periodically broadcasts an RIT Data Request command (hereafter, beacon) to announce that it is ready to receive data. Immediately after sending a beacon, the receiver switches to a listening state for a fixed duration defined by the RIT Data Wait Duration (DWD), during which it waits for an incoming data frame.

On the other hand, when an upper-layer application generates a transmission request, the sender node waits for a beacon from one of its potential receivers. Once a beacon is detected and determined to be from the intended receiver, the sender immediately transmits a data frame. A timeout mechanism, the RIT Tx Wait Duration (TWD), defines how long the sender waits for a beacon; if no beacon arrives within this interval, the transmission attempt is treated as a failure.

Compared with other asynchronous low-power MAC protocols, the receiver-initiated approach offers advantages in channel efficiency. For instance, CSL (Coordinated Sampled Listening) requires the sender to transmit a long wake-up frame sequence until the receiver wakes up. This results in significant control overhead and increased bandwidth usage.

In contrast, receiver-initiated protocols require only the reception of a beacon to start communication, eliminating the need for repeated wake-up frames. As a result, idle listening and control overhead remain low even in dense deployments.

Receiver-initiated MAC protocols offer energy-efficient asynchronous communication, but several structural limitations have been widely reported in the literature. These limitations arise directly from the beacon-driven communication model: multiple transmitters may respond to the same beacon, the beacon interval constrains delay and throughput, and performance varies significantly depending on traffic distribution and network topology. These issues have motivated numerous studies proposing enhancement mechanisms to improve contention avoidance, delay characteristics, and adaptability. The main structural challenges commonly discussed in prior work are summarized below.

### 2.2. Structural Challenges of Receiver-Initiated MAC Protocols

Receiver-initiated MAC protocols enable low-power and simple asynchronous operation, but they also exhibit several structural challenges arising from their basic communication mechanism:

- Sender Contention: Because multiple sender nodes may respond to the same beacon, contention around the beacon timing is likely to occur. Since the communication opportunity is restricted to the beacon event, sender-side collisions are more frequent than in conventional CSMA/CA-based approaches, especially under locally concentrated traffic.
- Energy–Delay Trade-off: A shorter beacon interval increases communication opportunities and reduces delay, but also raises the receiver’s energy consumption due to more frequent beacon transmissions. In contrast, a longer interval improves energy efficiency but reduces throughput and increases delay, while also increasing the probability of sender contention.
- Performance Variation under Different Topologies and Loads: In multi-hop networks, load tends to concentrate on relay nodes or nodes with high forwarding demand. As beacon waiting time accumulates, network-wide communication efficiency and fairness in energy consumption are negatively affected.
- Lack of Dynamic Adaptability: When the beacon interval is fixed, the protocol cannot flexibly adapt to temporal or spatial variations in traffic. In bursty traffic conditions, insufficient adaptation may lead to increased delay and a reduced success rate.

### 2.3. Evolution of Receiver-Initiated MAC Protocols

#### 2.3.1. Origins and Early Development of Receiver-Initiated MAC Protocols

The concept of receiver-initiated MAC protocols was first introduced by Lin et al. in 2004 with the proposal of RICER (Receiver-Initiated CyclEd Receiver) [5]. RICER demonstrated the basic idea of receiver-driven communication, where a receiver periodically emits a signal and a sender begins transmission upon detecting it. This work formed the foundation for later practical protocol designs.

A major step toward a usable receiver-initiated MAC was RI-MAC, proposed by Sun et al. in 2008 [6]. RI-MAC organized the receiver-initiated idea into a concrete and implementable protocol and became the basis for many subsequent studies. Similar to RICER, a receiver periodically broadcasts a beacon, and a sender transmits data immediately after detecting the beacon. RI-MAC also introduced several mechanisms to make this operation stable under realistic wireless conditions, including collision mitigation, delay reduction, and throughput improvement.

In particular, RI-MAC addressed the contention that occurs when multiple senders react to the same beacon by randomizing the beacon interval and embedding backoff-related information in the beacon to coordinate transmission timing. RI-MAC also supports consecutive frame exchange within a single communication opportunity, enabling more practical and efficient data transfer than simple one-packet exchanges. Through these mechanisms, RI-MAC maintains the basic structure of receiver-initiated communication while providing stability and extensibility suitable for real wireless network environments.

While RI-MAC introduced several practical mechanisms, a number of limitations have also been pointed out in later studies. For example, although the use of randomized backoff helps reduce contention, it increases the waiting time before data transmission and may lead to noticeable delays under heavy traffic. The fixed beacon interval also limits the ability to adapt to temporal or spatial variations in traffic load. In addition, in multi-hop or high-density networks, the increased density of beacon transmissions can raise channel occupancy, which becomes a scalability concern.

These issues identified in RI-MAC motivated many of the subsequent extensions of receiver-initiated MAC protocols and also influenced the later design direction of IEEE 802.15.4e RIT.

Around the same period, IRDT (Intermittent Receiver-driven Data Transmission) was proposed in Japan [7]. IRDT shares the basic receiver-initiated structure but follows a different design philosophy from RI-MAC. It is mainly intended for dense multi-hop mesh networks and adopts a cross-layer approach in which short control packets exchanged at the MAC layer are used to continuously update the status and routing-related information of neighboring nodes. Based on this information, a sender categorizes neighboring nodes by forwarding direction (e.g., forward or lateral) and opportunistically selects a currently available next hop. IRDT also performs a lightweight request–response exchange before data transmission, which localizes collisions to small control packets and improves robustness and throughput in multi-hop environments. Compared with RI-MAC, which focused primarily on low-power single-hop communication, IRDT is positioned as a mesh-oriented design that considers global network structure and load distribution, forming another line of receiver-initiated MAC research.

Building on these early protocols, research on receiver-initiated MACs has expanded in several directions, including collision reduction, energy savings, and improved adaptation to multi-hop topologies. Among these extensions, PW-MAC [8] is a representative approach that reduces the sender’s idle listening cost by predicting the receiver’s beacon timing.

#### 2.3.2. Predictive Beacon Scheduling

PW-MAC (Predictive-Wakeup MAC) [8] is a predictive receiver-initiated MAC protocol designed to significantly reduce the idle-listening cost on the sender side. In RI-MAC and IEEE 802.15.4e RIT, the sender must keep its radio on while waiting for a beacon from the intended receiver. When the receiver’s wake-up interval is long, this idle listening becomes the dominant source of energy consumption.

PW-MAC addresses this issue by allowing the sender to learn and predict the pseudo-random wake-up schedule of the receiver. The receiver shares its internal state (e.g., the seed of its pseudo-random sequence) during the initial communication, enabling the sender to activate its radio only at the predicted wake-up times. Correction information is exchanged only when the prediction error becomes large, so the additional overhead remains small while maintaining prediction accuracy.

However, because predicted wake-up slots may be shared among multiple senders, collisions can occur easily in dense networks, and the protocol becomes sensitive to contention and hidden terminals. To mitigate these issues, AS-PW-MAC (Adaptive Synchronous PW-MAC) [9] was introduced. AS-PW-MAC synchronizes beacon schedules among neighboring receivers to stabilize prediction accuracy and suppress the drift and error accumulation observed in PW-MAC. It also adjusts the wake-up schedule according to local traffic load and node density, reducing collision probability and improving reliability in dense deployments. Although this approach increases the overhead required to maintain synchronization, previous studies report that AS-PW-MAC provides more stable performance than PW-MAC in high-density network environments.

#### 2.3.3. Enhancements for Collision and Congestion Reduction

In receiver-initiated MAC protocols, multiple senders may react to the same beacon at the same time, which makes collisions more likely compared with sender-initiated schemes. This beacon-dependent structure is particularly sensitive to high-density deployments and Many-to-One traffic patterns, where simple backoff mechanisms are often insufficient. To address these issues, several enhanced protocols have been proposed.

RIVER-MAC [10] assumes that collisions are unavoidable in the moment immediately after beacon reception. When the receiver detects a collision, it transmits a short notification frame to guide the involved senders into a retransmission phase. By letting the receiver actively control the contention after a collision, RIVER-MAC achieves stable performance in heavy-load scenarios where many nodes contend for the same parent. However, this design increases the processing load on the receiver, and therefore assumes that the receiver has sufficient energy resources.

A-MAC [11] takes a different approach by estimating channel congestion based on past communication outcomes. Using this information, the protocol adaptively adjusts MAC parameters such as the beacon interval or transmission probability. When traffic is light, the protocol extends the wake-up interval to reduce energy consumption; under heavy traffic, it tightens the parameters to suppress collisions. Although this adaptive behavior improves flexibility, its effectiveness depends on the accuracy and stability of congestion estimation, and rapid traffic fluctuations may be difficult to follow.

REA-MAC [12] introduces a reservation-based mechanism to avoid sender-side collisions. Instead of allowing multiple transmitters to respond to the same beacon, the receiver explicitly selects one sender for the upcoming transmission opportunity, thereby improving reliability under high contention. On the other hand, this coordination step introduces additional overhead, which may reduce efficiency in light-load scenarios or when the payload size is small.

#### 2.3.4. IEEE 802.15.4e RIT

The IEEE 802.15.4e amendment introduced RIT (Receiver-Initiated Transmission) as a receiver-driven link establishment mechanism [1]. RIT generalizes earlier receiver-initiated MAC designs and defines them in a simplified and lightweight form. Consistent with the design principles of the IEEE 802.15.4 family, the MAC layer is strictly separated from upper-layer functions such as routing and traffic management. Therefore, unlike RI-MAC or IRDT, RIT does not include mechanisms for multi-hop forwarding, load balancing, or higher-layer coordination. Its functionality is limited to the minimal operations needed for 1-hop communication.

The 802.15.4e amendment was later integrated into the main IEEE 802.15.4–2015 specification [13], where RIT was incorporated as an official MAC option together with CSL (Coordinated Sampled Listening). Through this integration, RIT was moved from an extension profile into the primary standard, establishing it as a formal choice for low-power asynchronous MAC operation. The separation of MAC and upper layers was maintained in the revision, and RIT still provides only the basic receiver-initiated handshake without any mechanisms for multi-hop coordination.

As a result of this design policy, RIT preserves the core idea of receiver-initiated communication in a very compact form. While the lightweight structure is beneficial for energy efficiency and implementation simplicity, higher-layer issues—such as load concentration near the sink in multi-hop networks, sender contention, and the need for traffic-adaptive behavior—remain outside the scope of the standard. Thus, RIT alone cannot achieve network-level optimization, and additional mechanisms must be implemented at upper layers when applying RIT to practical multi-hop deployments.

#### 2.3.5. Practical Extensions of IEEE 802.15.4e RIT

F-RIT (Feathery-RIT) [14] is a practical receiver-initiated MAC protocol deployed in Japan for utility smart meters, such as gas and water systems under the U-Bus Air/Wi-SUN JUTA profile. F-RIT follows the basic operation of IEEE 802.15.4e RIT, but introduces several lightweight extensions designed for long-term battery-powered meter devices. These extensions aim to minimize frame size, reduce radio-on time, and suppress contention in low-traffic environments.

The main features of F-RIT are: (i) minimization of the RIT Data Request (beacon) frame, (ii) a short pre-transmission channel check (Pre-CS) that immediately aborts transmission when the channel is busy, and (iii) a simple acknowledgment sent after beacon reception to explicitly confirm link establishment. These mechanisms are effective for low-volume and low-frequency meter data collection, where reducing active radio time is essential. Experiments have reported that F-RIT maintains stable performance even in outdoor interference environments. However, because Pre-CS does not involve a backoff procedure, it may cause missed transmission opportunities under hidden-terminal conditions or in dense deployments, limiting its effectiveness in suppressing contention.

In addition, when F-RIT is applied to higher traffic loads or to bidirectional communication, a structural issue known as deafness becomes significant. When a node enters the “Tx Wait” state to receive a beacon from its parent, it temporarily stops transmitting its own beacons. As a result, child nodes may never receive a beacon and repeatedly experience transmission timeouts. Prior studies have shown that this phenomenon is a major cause of performance degradation under high load.

To address this issue, eF-RIT (Enhanced F-RIT) [15] introduces an interleaved beacon transmission mechanism. Even while waiting for a parent’s beacon, the node continues to maintain its internal RIT timer, and when the timer expires, it temporarily interrupts the waiting state to transmit its own beacon. This enables the reception of data from child nodes even during Tx Wait, effectively eliminating most of the timeout events observed in F-RIT. Because eF-RIT does not modify the frame format, compatibility with existing JUTA equipment is preserved, making it suitable for dense and bidirectional deployments in utility networks.

### 2.4. Limitations of Existing Receiver-Initiated MAC Research

Receiver-initiated MAC protocols have been widely studied as asynchronous, low-power communication schemes, and numerous enhancements and variants have been proposed over the past two decades. However, compared with other low-power wireless technologies—such as TSCH, BLE, and LoRaWAN—which have been broadly adopted in industrial deployments, receiver-initiated MACs have only limited real-world use. For small- to medium-scale sensing systems where delays on the order of tens of seconds are acceptable, the low-duty-cycle and asynchronous operation of receiver-initiated schemes can still be attractive, and recent studies have explored their applicability to energy-harvesting sensor devices [16]. A survey of existing literature and implementations suggests that, beyond the inherent technical challenges of the protocol structure, practical deployment has been hindered by inconsistencies in implementation details, evaluation methodologies, and platform support. This section summarizes these issues and clarifies the remaining technical and implementation-related limitations.

#### 2.4.1. Structural Congestion in Many-to-One and Multi-Hop Topologies

In receiver-initiated MAC protocols, communication opportunities are generated exclusively by periodic beacon transmissions from the receiver. This design yields high energy efficiency under low traffic, but limits the number of available transmission opportunities in many-to-one or multi-hop networks, where traffic naturally converges toward upper-layer nodes.

First, when multiple child nodes attempt to respond to the same beacon, synchronous contention occurs. Because transmission attempts are bound to the beacon timing, such contention is fundamentally difficult to avoid and can significantly reduce delivery performance in many-to-one scenarios.

In multi-hop networks, both data volume and waiting time accumulate at relay nodes. A relay node must handle its own outgoing data in addition to forwarded traffic from children, which increases beacon-waiting delays and results in reduced throughput or buffer overflows. Upper-layer nodes suffer the most congestion, increasing the likelihood of cascading performance degradation throughout the network.

#### 2.4.2. Lack of Cross-Layer Integration

Most studies on receiver-initiated MAC protocols focus primarily on MAC-layer behavior, and only a limited number consider systematic interactions with upper layers. In practical sensor networks, however, factors such as routing paths, data generation intervals, and traffic distribution at the network and application layers have a direct influence on MAC-layer operation. Optimizing the MAC layer alone therefore provides only partial insight into the overall system performance.

In receiver-initiated protocols, communication opportunities are strictly determined by beacon transmissions from the receiver. This structural characteristic means that routing decisions and traffic skew at upper layers directly affect contention levels, waiting times, and forwarding behavior at the MAC layer. Despite this tight coupling, existing studies rarely examine how different routing schemes or application workloads influence the protocol’s performance.

An exception can be found in IRDT, which adopts a cross-layer design that coordinates MAC behavior with routing decisions. IRDT demonstrates that receiver-initiated communication inherently possesses strong inter-layer dependencies, particularly in multi-hop mesh networks. In contrast, IEEE 802.15.4e RIT strictly adheres to the principle of layer separation. During standardization, RIT was intentionally limited to the minimum functionality required for one-hop communication, leaving multi-hop forwarding, load balancing, and adaptation entirely to upper layers. While this simplifies the MAC specification, it also restricts the protocol’s ability to optimize performance at the network scale.

These observations suggest that effectively leveraging the characteristics of receiver-initiated MAC protocols requires an integrated design that considers the MAC, routing, and application layers together. However, research approaches based on such a holistic perspective remain limited, and the lack of cross-layer integration remains one reason why receiver-initiated protocols have not gained broader practical adoption.

#### 2.4.3. Lack of Consistent Implementation and Evaluation Practices

Research on receiver-initiated MAC protocols often relies on simulation or hardware platforms for performance evaluation. However, an examination of existing literature shows that implementation details and evaluation environments vary widely across studies. This inconsistency makes meaningful comparison difficult.

Different implementation layers have been used in past work, including network simulators such as ns-2 [17], Cooja [18], and OMNeT++ [19], as well as hardware platforms based on TinyOS [20] or Contiki OS [21]. For example, RI-MAC has been evaluated on ns-2 and TinyOS, RIVER-MAC has been evaluated on Contiki and Cooja, and F-RIT has been tested on commercial Wi-SUN [22] devices. As a result, the code base and execution environment differ substantially across publications.

Basic MAC-layer behaviors also differ among studies. Beacon timing, backoff handling, the presence or absence of compact frames, and the implementation of continuous transmission are not standardized. Reference implementations are rarely provided, and many studies do not release their code, making reproducibility difficult.

Evaluation conditions vary as well. Topology design, radio propagation models, capture effects, the presence or absence of clock drift, and traffic generation intervals are not aligned across studies, and the impact of wireless conditions is often not analyzed in a unified manner. Such inconsistencies are a known issue in WSN research [23], and receiver-initiated MAC protocols are no exception.

Due to these differences, it is often unclear whether performance gaps reported in the literature arise from the protocol design itself or from differences in implementation and evaluation settings. The lack of common baselines makes cumulative progress and fair comparison difficult. For practical use and future development, standardized reference implementations and reproducible evaluation environments are essential.

### 2.5. Positioning of This Work

Although many improvements have been proposed for receiver-initiated MAC protocols, there is still no common reference implementation or unified evaluation environment. As a result, comparing different schemes and making design decisions has remained difficult. In this work, we implement an IEEE 802.15.4e RIT based receiver-initiated MAC protocol on ns-3 and build an evaluation environment where several contention avoidance mechanisms proposed in previous studies can be applied as modular options. This environment makes it possible to examine the operating conditions and design trade-offs of each mechanism under unified settings.

We adopt ns-3 for three main reasons. First, it can reproduce protocol behavior in an integrated manner across physical, MAC, network, and application layers. Second, the IEEE 802.15.4 stack in ns-3 has been actively extended, making it suitable for modeling low-power sensor networks in detail. Third, the implementation code can be published, which helps ensure reproducibility. These characteristics allow us to clarify the implementation details of receiver-initiated MAC protocols and provide a reusable evaluation framework.

The implementation process and the comparison of optional modules also reveal that receiver-initiated protocols, including RIT, have structural limitations that cannot be resolved by MAC-layer design alone. In particular, because communication opportunities depend on the receiver’s beacon schedule, load concentration becomes significant in multi-hop or Many-to-One topologies, and MAC-only improvements may not fully address this issue. This observation is consistent with the motivation behind cross-layer designs such as IRDT, which connects MAC and routing decisions.

The goal of this study is not to determine the superiority of specific techniques but to verify the fundamental components of receiver-initiated MAC protocols in a reproducible manner and to provide a common evaluation environment that future researchers can use when designing or selecting protocol configurations. The ns-3 implementation and evaluation platform developed in this work can serve not only for module-level comparisons but also as a foundation for future studies on cross-layer integration and multi-hop network design.

## 3. Implementaion in ns-3

This section describes the implementation of the Receiver-Initiated Transmission (RIT) mechanism [1], which is defined as a low-power MAC option in IEEE 802.15.4e, on the ns-3 network simulator.

The implementation extends the standard LrWpan model in ns-3. In addition to the basic RIT behavior, several contention avoidance mechanisms proposed in previous studies are implemented as optional modules that can be enabled or disabled. To support future extensions such as cross-layer designs and adaptive operation, key parameters are exposed as attributes so that they can be configured externally.

**Figure 2:**
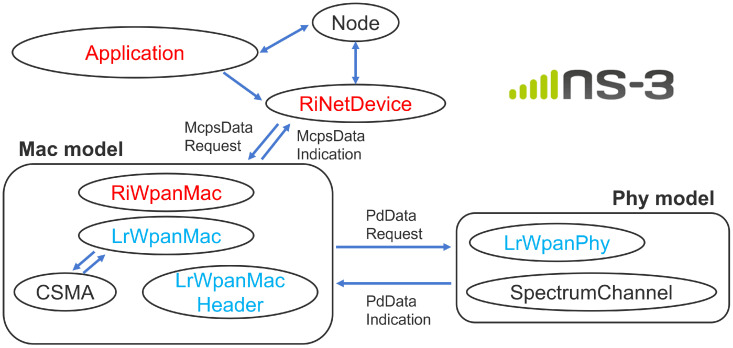
Class structure of the receiver-initiated MAC implementation in ns-3.

Figure 2 shows the class structure of the implementation. This design maintains compatibility with the existing protocol stack while allowing the required functions for receiver-initiated MAC operation to be incorporated in a localized and flexible manner. Some member functions and internal variables have been changed from “private” to “protected” or “public” to simplify inheritance and external access.

### 3.1. Mode Management

In a receiver-initiated MAC protocol, a node operates either as a sender or as a receiver depending on whether it has data to transmit. To represent these two roles explicitly, the implementation introduces “RitMacModeState” with two states: “SENDER_MODE” and “RECEIVER_MODE”. The main MAC-layer procedures branch according to this mode.

When an upper layer issues a transmission request, the node switches to “SENDER_MODE”. It waits for a beacon from the intended receiver and transmits a data frame once the beacon is detected. If the node is currently processing another transmission or reception and cannot switch modes immediately, the request is stored in an internal queue. The mode transition is evaluated again when the ongoing operation finishes.

When no transmission request is pending, the node remains in “RECEIVER_MODE”. It periodically sends RIT beacons and then enters the short data-wait window (DWD) to receive data from child nodes.

This mode-based design enables a clear separation between sender-side and receiver-side operations and allows the behavior of receiver-initiated MAC protocols to be accurately reproduced in ns-3.

### 3.2. Beacon Transmission: "PeriodicRitDataRequest()"

A node operating in “RECEIVER_MODE” periodically transmits a beacon (RIT Data Request Command) to announce that it is ready to receive data from nearby sender nodes. After a beacon is generated, it is immediately passed to the PHY layer for transmission. The next beacon event is then scheduled based on the predefined beacon interval (“macRitPeriod”).

If the node is unable to transmit a beacon—for example, because it is currently handling a transmission or reception—the beacon for that cycle is skipped.

### 3.3. Transmission Requests from Upper Layers

In this implementation, transmission requests from upper layers are accepted independently of the current MAC state and are stored in a transmission queue. When a request arrives and the node is neither transmitting nor receiving, the node attempts to switch to “SENDER_MODE” immediately. If an ongoing operation prevents an immediate mode transition, the request remains in the queue and is reconsidered after the current operation finishes.

The decision to transition into sender mode is evaluated at the following three points:

- Immediately before periodic beacon transmission (“RECEIVER_MODE”)
- Right after finishing “SENDER_MODE”
- Right after finishing “RECEIVER_MODE”

### 3.4. Data Frame Transmission

When a node enters “SENDER_MODE”, it waits for a beacon from its intended receiver and starts data transmission once the beacon is detected. During this phase, the node enters a waiting state and monitors the channel for the duration specified by the Tx Wait Duration (TWD). If no beacon is received within this interval, the attempt is treated as a timeout and the transmission fails.

If a beacon is detected, the beacon is passed to the upper layer for destination checking. If the beacon originates from the intended receiver, the node sets the sender address contained in the beacon as the destination of the data frame and immediately begins transmission.

### 3.5. Receive Completion Callback: “PdDataIndication()”

Events issued by the PHY layer must be handled differently depending on whether the node is operating as a sender or a receiver. Therefore, this function explicitly branches its behavior based on the current “RiMacModeState”. Each branch performs the appropriate processing required for the corresponding mode.

#### 3.5.1. Reception of Data Frames

Data frame processing is performed only when the node is in “RECEIVER_MODE”. If the destination address contained in the received frame matches the local node, the frame is delivered to the upper layer. If the address does not match, the frame is discarded as it is intended for another node.

#### 3.5.2. Reception of Beacon Frames

Beacon frames are meaningful only for sender-side behavior because RIT uses beacons as the sole trigger for data transmission. Therefore, beacon handling is executed only when the node is in “SENDER_MODE”. After a beacon is received, it is passed to the upper layer for destination checking. If the beacon belongs to the intended receiver, the node generates a data frame and starts transmission. When the node is in "RECEIVER_MODE", beacon frames are ignored because they do not correspond to receiver-side processing.

#### 3.5.3. Reception of ACK Frames

ACK frames are processed only when the node is in “MAC_ACK_PENDING”. If a matching ACK is received, the corresponding data transmission is considered successful and the node returns to “RECEIVER_MODE”. If the MAC configuration does not require ACKs, ACK frames are ignored.

### 3.6. Transmission Completion Callback: “PdDataConfirm()”

This function is called when the PHY layer notifies the MAC of a transmission completion event. Depending on whether the transmission succeeded or failed, the MAC state is updated accordingly. In either case, the MAC state returns to “MAC_IDLE” after the processing is finished.

#### 3.6.1. Successful Transmission

(a) “SENDER_MODE”: If a data frame is transmitted successfully and ACKs are not required, the node immediately returns to “RECEIVER_MODE”. If ACKs are required, the node transitions to “MAC_ACK_PENDING” and waits for the ACK.
(b) “RECEIVER_MODE”: After a beacon is transmitted, the receiver must enter a short listening phase (DWD) to accept responses from child nodes. When this waiting period starts, a reception timeout (TWD) is also scheduled for the upper layer. The node then transitions to the receive state.

#### 3.6.2. Failed Transmission

(a) “SENDER_MODE”: When a data transmission fails, the event is reported to the upper layer, which decides whether to retry. If no retransmission is requested, the pending transmission request is discarded and the node returns to the listening state to wait for the next beacon.
(b) “RECEIVER_MODE”: If a beacon transmission fails, the receiver does not attempt retransmission. Instead, it immediately transitions to the sleep state.

### 3.7. Timeout Event

Timeout handling is required in receiver-initiated protocols to detect failures in transmission and reception, as well as to determine when a waiting period has expired. In this implementation, two timeout handlers are used: “ReceiverCycleTimeout()” for “RECEIVER_MODE” and “SenderCycleTimeout()” for “SENDER_MODE”.

#### 3.7.1. Receiver Timeout: “ReceiverCycleTimeout()”

This function is executed in “RECEIVER_MODE” when no data frame is received within the waiting period after a beacon transmission. If the MAC state at the moment of timeout is “MAC_SENDING” (i.e., the node is still transmitting an ACK), the timeout is extended because the communication is considered ongoing. If there is a pending transmission request, the node transitions to “SENDER_MODE”. Otherwise, it moves to the sleep state.

#### 3.7.2. Sender Timeout: “SenderCycleTimeout()”

This function is executed in “SENDER_MODE” when no beacon is detected and no data is transmitted during the designated TWD interval. If the MAC state is “MAC_SENDING” or “MAC_ACK_PENDING”, the timeout is extended because a transmission is still in progress. If not, the transmission request is canceled, and any unsent packet is optionally returned to the sending queue. The node then transitions back to “RECEIVER_MODE”.

#### 3.7.3. Cancellation of Timeout Events

ns-3 is an event-driven simulator. All state transitions and operations are executed as scheduled events. The timeout events “ReceiverCycleTimeout()” and “SenderCycleTimeout()” are registered to enforce the maximum waiting time in their respective modes. When the corresponding communication process completes successfully before the timeout, the associated event is canceled to avoid unnecessary execution.

### 3.8. Routing

**Figure 3:**
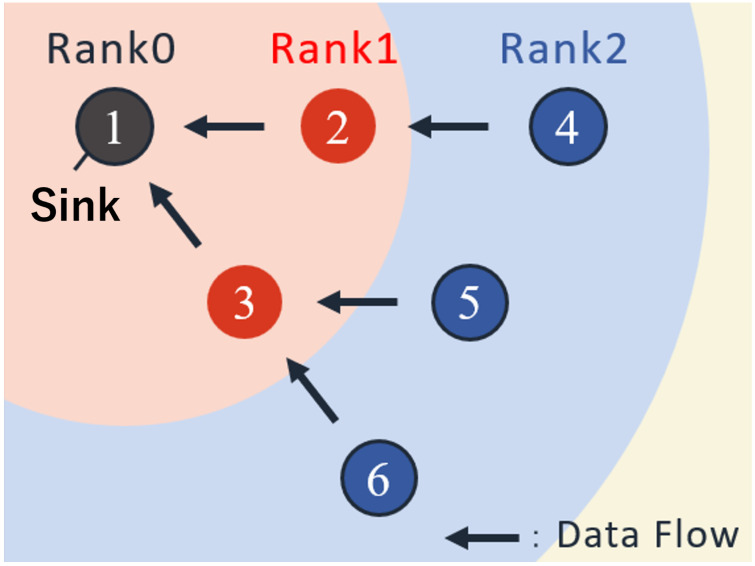
Example of Rank-Based Routing.

In this study, we focus on upward convergecast communication, where all data generated by sensor nodes is eventually delivered to the sink. Multi-hop forwarding is assumed at the MAC layer. Each node forwards packets through upper-layer nodes based on its rank, which represents the hop distance to the sink. Figure 3 illustrates an example of convergecast communication enabled by rank-based routing.

This routing mechanism leverages characteristics of receiver-initiated communication. Each beacon embeds the sender’s rank, and transmitting nodes use the received rank information to determine whether to forward data. Specifically, a node transmits only to neighbors with smaller rank values (i.e., closer to the sink). This opportunistic selection allows dynamic path formation without maintaining a routing table. Such a design is well aligned with receiver-initiated MAC behavior and has been examined in prior work [24].

For the purpose of evaluating multi-hop operation in this simulation, data forwarding is restricted to nodes whose rank differs by exactly one.

## 4. Modular Implementation of RIT Enhancement Options in ns-3

This section describes the implementation of several contention-avoidance mechanisms on top of the IEEE 802.15.4e RIT–based receiver-initiated MAC developed in the previous chapter. The purpose of these extensions is to provide a unified platform where existing enhancement ideas can be examined under consistent conditions.

The mechanisms selected for this study are based on ideas proposed in two important receiver-initiated MAC protocols: RI-MAC, which established the early conceptual foundation of receiver-initiated communication, and F-RIT, which was designed for practical deployment in utility metering systems. Among the techniques suggested in these works we selected a set of features that remain compatible with the fundamental RIT operation, can be integrated without modifying the core procedure, and offer modular on/off control for comparative evaluation.

These enhancement modules are implemented as optional components that can be enabled independently. This chapter documents their design and integration and prepares a common implementation basis for evaluating their behavior in later sections.

### 4.1. Selectable Channel Access Mechanisms

This study provides three selectable channel access mechanisms for both beacon transmission and data transmission. Each mechanism is implemented as an independent option that can be switched on or off in the simulation.

- CSMA/CA: The standard IEEE 802.15.4 method. A transmission is controlled by a combination of random backoff and CCA, and the sender retries when CCA fails until the retry limit is reached.
- Pre-CS: A lightweight carrier sensing method proposed in F-RIT. Only a single CCA is performed immediately before transmission, and no backoff or retry is executed. If the CCA fails, the node abandons the transmission opportunity.
- No Carrier Sense: CCA is skipped entirely and the frame is transmitted immediately. This option is mainly used for small frames such as ACKs where quick response is required.

The Pre-CS method is designed to reduce the energy cost of beacon transmission and to prioritize data traffic. The basic assumption is that a CCA failure before beacon transmission indicates that a nearby node is already performing a data communication, and forcing a beacon in such a situation increases the risk of collision. Therefore, the Pre-CS mechanism does not retry the beacon and simply waits for the next beacon interval, reducing both contention and unnecessary energy consumption.

F-RIT was evaluated through real deployments, where the wireless environment was assumed to be relatively stable. Based on this assumption and the requirement to comply with radio regulations, Pre-CS was also applied to data transmissions in order to improve reliability.

However, Pre-CS was originally designed mainly for beacon transmission. Since only a single CCA is performed and the transmission is abandoned immediately when the CCA fails, the method reacts sensitively even to short-term interference. As a result, a data transmission opportunity may be lost even in situations where a small timing shift would allow a successful transmission. This characteristic can lead to reduced communication success rates when Pre-CS is applied to data frames.

In contrast, when carrier sensing is skipped and the sender transmits immediately, the sender can assume that the channel near the receiver is temporarily free because the sender has just received the receiver’s beacon correctly. In such cases, transmitting without CCA may avoid contention. However, applying this approach to beacon transmission is not desirable because frequent beacons would increase channel occupancy and interfere with data communications from other nodes.

When CSMA/CA is used for data transmission, multiple backoff attempts can extend the transmission process. If the receiver’s DWD (Data Wait Duration) is short, this extended backoff may cause the data frame to miss the acceptable timing window, leading to transmission failures. Therefore, when CSMA/CA is used, the relationship between the DWD setting and the transmission duration must be carefully considered.

It should also be noted that in some countries or regions, performing CCA is required by radio regulations. Such legal requirements must be taken into account when selecting a channel access mechanism.

### 4.2. Beacon ACK

This option introduces a mechanism in which a sender does not transmit its data immediately after receiving a beacon. Instead, the sender first replies with a short acknowledgment frame. This design is based on the implementation used in F-RIT.

When Beacon ACK is enabled, a sender that receives a beacon transmits a short ACK frame to the beacon source. This ACK explicitly indicates the sender’s intention to communicate and confirms link establishment. Since the ACK frame is short and fixed in length, the receiver can quickly determine whether a sender intends to transmit data. This makes it possible to shorten the Data Wait Duration (DWD) to match the ACK duration.

In applications where the data frame size varies, the use of Beacon ACK eliminates the need to allocate a long DWD based on the maximum possible frame size. As a result, the energy consumption associated with waiting for incoming data can be reduced. When CSMA/CA is used for data transmission, Beacon ACK also allows the DWD to be shortened, since the timing uncertainty introduced by backoff is reduced before data transmission begins. However, the ACK itself introduces an additional transmission opportunity, which may slightly increase interference and reduce the communication success rate in dense deployments.

Beacon ACK aims to balance power efficiency and communication stability. Its effectiveness depends on the network density and the expected traffic load, and therefore requires careful configuration.

### 4.3. Beacon Interval Control (Fixed or Randomized)

In this option, the interval at which a receiver transmits its beacon can be selected from two modes.

- Fixed Interval: The beacon is always transmitted at a constant period. This is the standard behavior defined in IEEE 802.15.4e RIT.
- Randomized Interval: Proposed in RI-MAC. The beacon interval is varied randomly within the range of 0.5 to 1.5 times a predefined base value.

The randomized interval prevents unintended synchronization among neighboring nodes. This reduces beacon collisions and sender-side contention. As a result, it can mitigate channel congestion, stabilize beacon detection, and improve fairness among nodes.

This effect is particularly useful in applications with periodic data generation, such as environmental sensing. In such applications, node activity tends to align naturally and may lead to local synchronization. This synchronization can trigger bursts of contention and frame collisions. Introducing variability in the beacon interval helps avoid these risks.

However, interval randomness may also cause uneven distribution of communication opportunities. This can lead to missed beacons and eventual transmission timeouts. The impact becomes more noticeable in low-density networks, and the benefit of randomization is limited when the traffic load is light.

### 4.4. Compact Beacon Frame

This option introduces a reduced-size beacon format for the periodic beacons transmitted by receiver nodes in RIT. The method is based on the design used in F-RIT and aims to reduce the overhead of beacon transmission, which accounts for a significant portion of network-wide energy consumption.

In the standard MAC frame format, fields such as the PAN identifier and extended information elements are included. However, in RIT communication, the sender only needs to identify the address of the receiver to respond. These additional fields are therefore not essential. To minimize the frame size, the beacon is simplified to include only the receiver’s address, restricted to the 2-byte short address format. As a result, the total frame length is reduced to 14 bytes, including the frame control field and the FCS.

This reduction decreases transmission time, energy consumption, and channel occupancy. It is particularly effective in high-density networks or under concentrated traffic, where shorter beacons help reduce collision probability and improve overall communication efficiency.

Given these advantages, this compact beacon format is adopted as the default configuration in our implementation.

## 5. Simulation Setup

This section describes the components required to execute scenario-based simulations of the receiver-initiated MAC protocol implemented in this study. The goal is to evaluate protocol behavior under realistic network conditions by incorporating clock drift, node placement, traffic models, and practical parameter settings.

### 5.1. Clock Drift

In practical sensor nodes, the local clock is affected by oscillator inaccuracy and environmental variations, resulting in small but non-negligible timing errors. To model this behavior, the elapsed sleep duration is adjusted using a frequency skew and random noise. For an ideal elapsed time *t*, the corrected sleep time $T_{\mathrm{sleep}}$ is calculated as

(1) $$T_{\mathrm{sleep}}=t\left(1+\delta \right)+\epsilon_{t}$$

where δ denotes the frequency skew and $\epsilon_{t}∼N\left(0,Kt\right)$ represents Gaussian noise with variance proportional to *t*.

This correction is applied only when scheduling the next sleep period, causing each node to wake up at slightly different times. As a result, the simulation reproduces realistic timing variations typically observed in low-cost sensor hardware.

### 5.2. Network Topology

**Figure 4:**
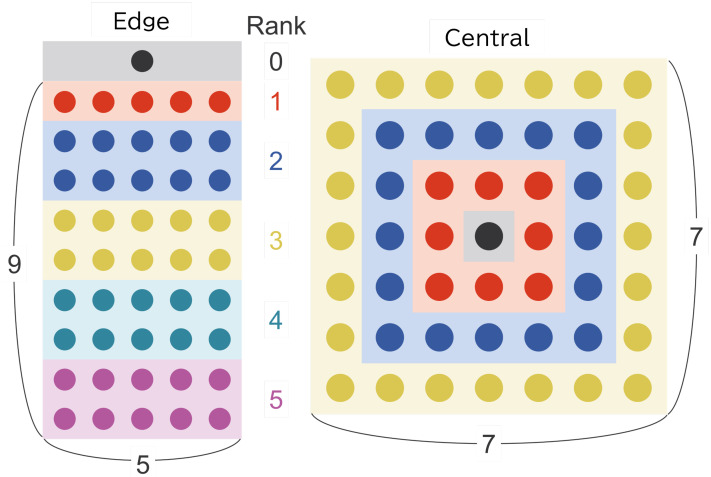
Node placement patterns (Edge and Central configurations).

To investigate how node placement affects channel contention and interference at the MAC layer, two types of network topologies were designed. The layouts of these topologies are illustrated in Figure 4. Router nodes are arranged in a grid, and the position of the sink node and the inter-node spacing are varied to create environments with different contention characteristics. Note that the assumptions regarding communication coverage and interference, which form the basis for the topology design, are described in the simulation setup presented in Section 5.4.

- Edge: The sink node is placed 50 m outside the edge of the grid so that five Rank 1 nodes remain within its communication range. The maximum rank is set to five, representing a medium-scale multi-hop network with multiple forwarding stages. Router nodes are placed at 25 m intervals, producing significant coverage overlap and frequent channel interference. The number of Rank 1 nodes is limited to five in order to avoid excessive overhead around the sink.
- Central: The sink node is located at the center of the grid, and all router nodes are placed uniformly at 50 m spacing. In this configuration, many nodes may respond to the sink’s beacon simultaneously, increasing the likelihood of contention and hidden-terminal effects. This topology is intentionally designed to emphasize interference and to evaluate the robustness of MAC-layer contention-avoidance mechanisms.

### 5.3. Application Model

In this study, we designed an application model in which each node generates data either at fixed intervals or at irregular timings. Two types of traffic patterns are considered:

- Periodic Transmission: Each node sends data at a constant interval. This pattern represents applications such as environmental monitoring or periodic status reporting.
- Random Transmission: Each node determines its transmission interval based on a uniform random distribution, resulting in irregular data generation. This pattern is intended to model event-driven applications or traffic with occasional bursts.

At the beginning of each simulation run, the initial transmission time of every node is randomly selected from a uniform distribution. This avoids the situation where many nodes start sending packets simultaneously due to identical initial phases.

### 5.4. Input Parameters and Simulation Settings

Table 1 summarizes the main parameters used in the simulation. Each parameter is chosen to reflect conditions commonly observed in practical low-power sensor networks.

In the simulation, wireless signal propagation is modeled using the default LogDistancePropagationLossModel provided by ns-3, and propagation delay is modeled using the default ConstantSpeedPropagationDelayModel.

Under the employed propagation model and the receiver sensitivity settings of the lr-wpan PHY, the maximum communication range can be estimated to be on the order of approximately 100 m. This value represents the upper bound at which packet reception is physically possible in the considered setting and is used as a reference when designing the network topology. Also, interference in the ns-3 lr-wpan PHY is not modeled using a simple distance-based range but is evaluated as the superposition of simultaneously received signals.

The data generation interval depends on the application type. For periodic traffic, each node generates a measurement every 300 s, assuming environmental monitoring tasks. For random traffic, transmission intervals are uniformly sampled between 180 and 600 s, representing sporadic events or anomaly detection.

Sensor data payloads are fixed at 8 bytes. RIT-specific parameters—such as the beacon transmission interval (“macRitPeriod”) and timeout durations—are configured as fixed values so that the impact of each MAC option can be evaluated clearly. When the Beacon-ACK option is enabled, the short waiting time after beacon transmission is set to 2 ms, as its duration is fixed in that configuration. Clock drift is modeled assuming sensor nodes that employ RC oscillators instead of crystal oscillators. A frequency skew of ±250 ppm is assigned from a uniform distribution, simulating realistic timing variations of low-cost nodes. To reduce simulation overhead, drift is applied only when scheduling the next beacon event.

The initial transmission times of the application layer and the initial RIT beacon times are randomized within the range determined by their respective parameters. This prevents phase alignment among nodes at the beginning of the simulation.

ACK processing is enabled for all packets. If an ACK is not received or a CCA failure occurs, the packet is immediately regarded as failed and dropped. Retransmissions above the MAC layer are not performed.

Each simulation runs for 1 day, which refers to the simulated time in ns-3. This duration was selected to be long enough to obtain stable averaged results in the presence of random factors, while keeping the simulation execution time practical. Also, to further reduce the impact of randomness, each configuration is evaluated using 20 runs with different random seeds, and the average results are used as performance metrics.

### 5.5. Use of Generative AI

In this study, generative AI tools ChatGPT (GPT-4.5 and GPT-5.1) and Google Gemini (Gemini 2.5 Flash and Gemini 2.5 Pro) were used under the full supervision of the authors for:

- assisting in organizing information from previous studies during the literature review,
- generating auxiliary Python scripts for parsing and visualizing simulation logs.

The AI tools were not used to generate scientific concepts, design experiments, analyze results, or draw any scientific conclusions. All technical content and interpretations were produced and validated entirely by the authors.

## 6. Simulation Results and Discussion

### 6.1. Evaluation Metrics

Table 2 summarizes the evaluation metrics used in this study.

In this study, the simulation results are visualized using heatmaps in order to compare the performance of different configuration settings. The correspondence between the horizontal-axis labels and the scenario conditions is summarized in Table 3. The vertical-axis labels and their mapping to CSMA configurations and ACK settings are shown in Table 4.

### 6.2. PDR

In the following results, BI denotes the beacon interval of the RIT MAC protocol. BI5 and BI3 correspond to beacon intervals of 5 s and 3 s, respectively.

**Figure 5:**
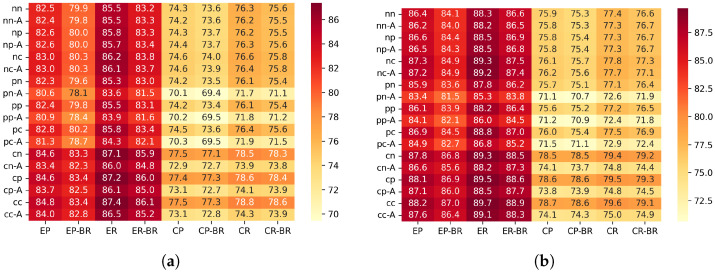
PDR (%) under BI5 and BI3 configurations. (**a**) BI5. (**b**) BI3.

Figure 5 show the PDR for each configuration.

#### 6.2.1. Differences Between Scenarios (PDR)

In the edge placement scenario, the random traffic generation pattern achieved a 2.3–4.0% higher PDR compared with the periodic generation pattern. This is because the edge placement has a larger number of hops, and transmission requests tend to concentrate on the upper nodes directly below the sink. With periodic generation, multiple nodes are likely to enter the transmission mode at the same timing, which increases contention and timeouts. In contrast, under random generation, transmission requests are temporally distributed, which helps balance the processing load on upper nodes and reduces collisions, resulting in improved PDR.

In the center placement scenario, random generation also improved the PDR by about 1.6–2.7%. Since the center placement has higher node density and shorter hop distances, the load is less likely to concentrate on a specific upper node. Therefore, although the improvement is smaller than in the edge scenario, the temporal distribution of requests still helps reduce contention, leading to a modest increase in PDR.

#### 6.2.2. Effectiveness of Beacon Randomization (PDR)

**Figure 6:**
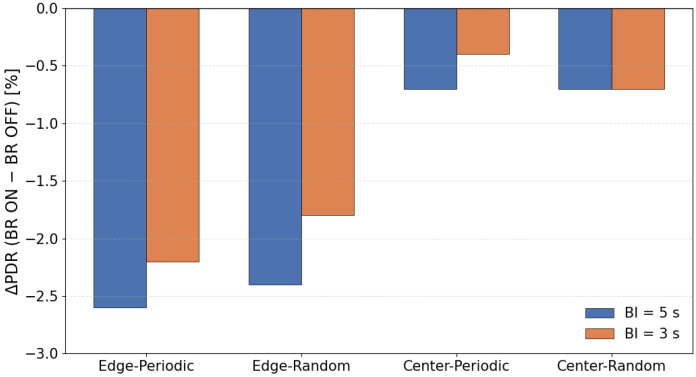
Difference in PDR (%) with and without Beacon Randomization.

To clarify the overall tendency of the impact of beacon randomization, Figure 6 illustrates the average PDR difference between configurations with and without beacon randomization.

In this evaluation, applying beacon randomization (“-BR”) significantly degraded the PDR, especially in the scenario where the sink node was placed at the edge of the network. On the other hand, when the sink was placed at the center, the degradation caused by BR was relatively small, and the difference from the non-randomized configuration was almost negligible. This tendency was observed not only in periodic traffic but also in random traffic generation.

Based on the protocol structure and the characteristics of the topology, this behavior can be interpreted as follows. In our implementation, a sender transmits DATA only when it receives a beacon from the parent node, and MAC-layer retransmissions are not performed. Therefore, when the beacon interval is fixed, the service interval at each hop is also fixed, and even if packets accumulate at upper hops, they are processed at relatively regular intervals. In contrast, when the beacon interval is randomized, the service interval becomes uneven, and during periods where the interval becomes longer, packets can temporarily accumulate near upper hops. As a result, multiple nodes are more likely to react to the same beacon at the same time, increasing sender contention.

In the edge placement scenario, multi-hop forwarding causes traffic to converge onto a single path toward the sink. Under these conditions, the combination of uneven service intervals and load concentration increases the likelihood that multiple nodes will respond to the same beacon simultaneously, leading to sender contention. When several nodes attempt to transmit within the same DATA Window (DWD), and since no MAC-layer retransmissions are performed in this implementation, such collisions directly appear as noticeable PDR degradation.

In contrast, in the center placement scenario, traffic becomes spatially distributed, and excessive load is less likely to concentrate on a single upper link. For this reason, even if the service interval becomes uneven due to beacon randomization, sender contention does not grow significantly, and the difference in PDR between BR and the non-randomized configuration remains small.

When the beacon interval was shortened from 5 s to 3 s, the degradation in PDR was greatly reduced. A shorter interval increases the total number of receiving windows, which lowers the probability that multiple nodes will concentrate on the same receiving window. In other words, increasing the number of transmission opportunities helps distribute instantaneous contention and suppresses immediate packet loss due to collisions.

Originally, beacon randomization is designed to prevent unintended synchronization among multiple receivers, which could otherwise cause excessive concentration of communication timing. However, under the evaluation conditions of this study—single-sink configuration, low-density topology, low traffic load, and small clock drift—there were few opportunities for such synchronization problems to occur. On the other hand, the variation in service interval caused by randomization tends to create temporary backlog and sender contention around upper hops. Therefore, the PDR degradation observed in this study can be interpreted as a result of the service-interval fluctuation exposing topology-dependent contention, rather than beacon randomization providing its expected desynchronization effect.

#### 6.2.3. Effectiveness of Beacon ACK (PDR)

**Figure 7:**
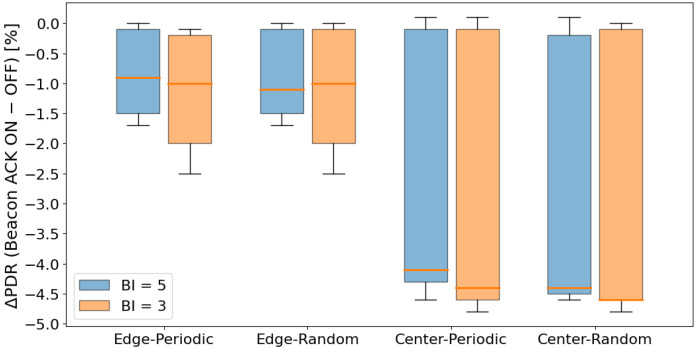
Difference in PDR (%) with and without Beacon ACK.

Figure 7 shows the distribution of the PDR difference between configurations with and without beacon ACK for each scenario.

In this evaluation, configurations that use a beacon ACK showed a tendency to have lower PDR when some form of CSMA processing is applied during data transmission, compared with configurations without a beacon ACK. This can be explained by the fact that sending a beacon ACK increases the total number of transmissions in the network, and the ACK itself becomes a new source of contention, while at the same time it does not contribute to reducing sender contention. In addition, in our implementation, even if contention occurs between the beacon ACK and the following data frame, the subsequent data transmission is not canceled, which results in unnecessary transmissions and further increases the chance of packet loss.

Looking at each scenario, the decrease in PDR in the Edge scenario was relatively small, and in many cases remained below 1%. In contrast, in the Center scenario, a degradation of about 4.1–4.5% was observed. This indicates that in high-density topologies, the increased transmission activity caused by ACKs becomes more noticeable and has a stronger negative impact.

On the other hand, in configurations where no CSMA processing is used for data transmission, almost no performance degradation was observed when applying beacon ACK. This behavior is likely due to the transmission sequence in which a node immediately sends a beacon ACK after receiving the beacon, and then continues directly to data transmission. In this case, from the perspective of surrounding nodes, the ACK and the subsequent data frame appear as one continuous transmission period. Because other nodes detect the channel as continuously busy using CCA, they cannot start a new transmission attempt. As a result, even though the beacon ACK slightly changes the timing of transmissions, actual contention does not increase, and the PDR remains mostly unaffected.

In contrast, when CSMA processing is used for data transmission, CCA and backoff must be executed after sending a beacon ACK. During CSMA processing, the channel is observed as idle, which makes it easier for other nodes to start preparing for transmission at the same timing. This increases the chance that multiple nodes will begin transmission simultaneously. Especially in situations where a mild synchronization exists due to the beacon cycle, multiple nodes tend to execute CCA nearly at the same time, resulting in more contention. In other words, the short waiting time inserted by CSMA breaks the continuous channel occupation created by the ACK, and this effect induces the performance degradation observed when beacon ACK is applied.

#### 6.2.4. Effectiveness of Channel Access Mechanisms for Data Transmission

In configurations where CSMA/CA is not applied on the sender side, the PDR reached a plateau and converged to similar values across all settings. This is because such configurations cannot avoid sender contention when multiple nodes react to the same beacon and attempt to transmit simultaneously.

In contrast, configurations that apply CSMA/CA showed improved PDR. When several nodes respond to the same beacon, the random backoff procedure allows only one node to eventually obtain the transmission right. This behavior helps avoid part of the collisions that would otherwise occur, leading to a tendency for the PDR to improve.

These results indicate that CSMA/CA during data transmission is effective in mitigating collisions caused by sender contention, especially in situations where many nodes tend to react to the same beacon timing. However, it should be noted that CSMA/CA does not fundamentally resolve the underlying cause of sender contention. It only reduces its impact. Therefore, achieving even higher transmission success rates in the future will require approaches that suppress the structural factors that create sender contention in the first place.

#### 6.2.5. Effectiveness of Channel Access Mechanisms for Beacon Transmission

In this evaluation, switching the channel access mechanism for beacon transmission—between CSMA/CA, Pre-CS, and no CCA—resulted in almost no noticeable differences in PDR. This suggests that beacon collisions were not a major performance constraint under our simulation conditions.

First, the evaluation environment used a single-parent topology with relatively low network load, small node density, and small clock drift. Under such conditions, beacon transmission timings rarely overlapped naturally, meaning that the collision-avoidance effect of CCA had limited opportunities to appear.

In addition, under the traffic conditions used in this study, the primary cause of communication failure was sender contention, where multiple nodes reacted to the same beacon due to traffic concentration at upper hops. Beacon-to-beacon collisions were not dominant. Therefore, changing the channel access mechanism for beacon transmission did not produce differences large enough to affect the overall success rate.

Furthermore, beacon frames are very short, and their physical transmission time is extremely small. Even when CSMA/CA is enabled, there is little margin for collision avoidance. Because of this structural characteristic, the behavior during contention is similar across different channel access mechanisms, resulting in only small observable differences.

Overall, these results indicate that, in this environment, contention on the data transmission side—rather than beacon-side collisions—dominates performance. This is consistent with the nature of receiver-initiated MAC protocols. It also implies that the impact of the channel access mechanism for beacon transmission may become significant only in higher-density or higher-load environments.

#### 6.2.6. Throughput

As a reference metric, throughput is presented to complement the PDR analysis. The throughput is derived from the PDR and represents the amount of application data successfully delivered to the sink per unit time. Throughput is calculated as

(2) $$T=\mathrm{PDR}\times N_{\mathrm{tx}}\times \frac{L_{\mathrm{payload}}}{I_{\mathrm{gen}}}$$

where PDR is the packet delivery ratio, $N_{\mathrm{tx}}$ is the number of transmitting nodes, $L_{\mathrm{payload}}$ is the application payload size (8 bytes), and $I_{\mathrm{gen}}$ is the mean packet generation interval (300 s for periodic traffic and 390 s for random traffic).

**Figure 8:**
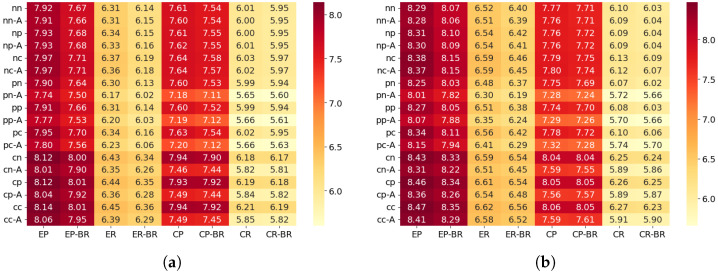
Throughput (bps) BI5 and BI3 configurations. (**a**) BI5. (**b**) BI3.

Figure 8 shows the derived throughput results under the BI5 and BI3 configurations, while Table 5 summarizes the theoretical maximum throughput for each scenario, corresponding to the case where PDR=100%. In this study, throughput is presented as a reference metric to translate the PDR results into an application-level data delivery perspective.

### 6.3. Wakeup Ratio

**Figure 9:**
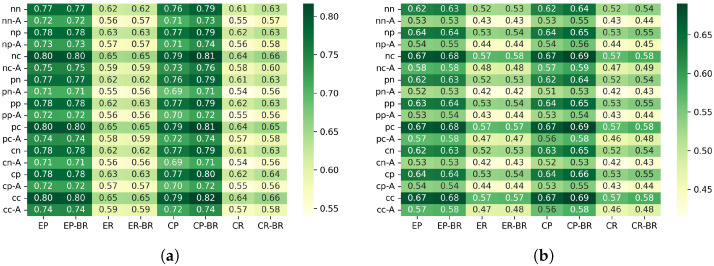
Wakeup Ratio (%) under BI5 and BI3 configurations. (**a**) BI5. (**b**) BI3.

Figure 9 show the average awake ratio for each configuration.

#### 6.3.1. Differences Between Scenarios (Wakeup Ratio)

The average awake ratio shows tendencies similar to those observed in the PDR results. In the Edge scenario, the random traffic generation pattern slightly reduced the awake ratio compared with the periodic pattern, and shortening the beacon interval from 5 s to 3 s resulted in a reduction of about 0.13–0.28%. This can be interpreted as a result of temporal dispersion of transmission requests under random generation, which reduces collision frequency near upper hops and consequently decreases unnecessary waiting and timeouts, leading to a shorter awake duration.

In the Center scenario, the random generation pattern also reduced the awake ratio by approximately 0.13–0.18%. Since the Center scenario has fewer hops and is structurally less prone to load concentration, the effect was not as large as in the Edge scenario. However, the reduced contention due to temporal dispersion of traffic still contributed to lower awake time.

Unlike the PDR results, the amount of reduction in awake ratio was nearly the same in both the Edge and Center scenarios. The awake ratio directly reflects each node’s waiting and receiving duration, and therefore is less affected by localized congestion at specific hops compared with PDR. As a result, the overall contention reduction caused by the temporal dispersion of traffic appeared evenly across scenarios, rather than being strongly influenced by structural differences.

#### 6.3.2. Effectiveness of Beacon Randomization (Wakeup Ratio)

In all scenarios, the configuration with randomized beacon timing showed a clear increase in the average awake ratio compared with the non-randomized configuration. This is because variations in the beacon interval cause nodes to spend more time waiting in the transmission mode. Since RIT allows data transmission only immediately after a beacon, if the beacon arrival is delayed, nodes that are already ready to transmit must remain awake until the next beacon. As a result, the awake time tends to become longer.

Therefore, under the evaluation conditions of this study, beacon randomization did not provide benefits in terms of awake time. Instead, the increased waiting time caused by irregular beacon arrivals dominated the behavior, leading to higher awake ratios.

#### 6.3.3. Effectiveness of Beacon ACK (Wakeup Ratio)

**Figure 10:**
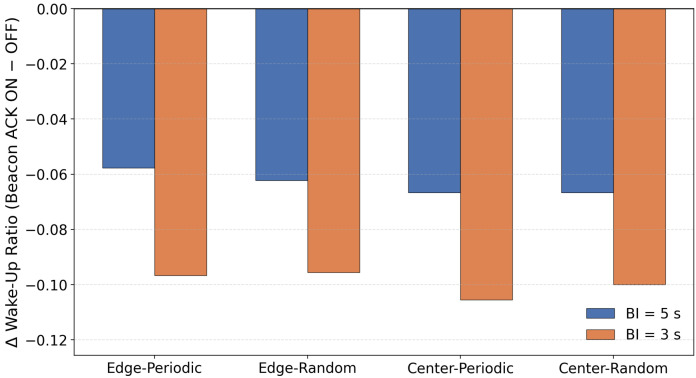
Difference in wakeup ratio (%) with and without beacon ACK.

Figure 10 shows the distribution of the Wakeup ratio difference between configurations with and without beacon ACK for each scenario.

In the configuration where beacon ACK responses are enabled (“-A”), the average awake ratio decreased by approximately 6–11% across all scenarios. This indicates that the ACK mechanism effectively shortens the waiting duration for data reception after each beacon, resulting in a noticeable reduction in awake time.

### 6.4. End-to-End Latency

**Figure 11:**
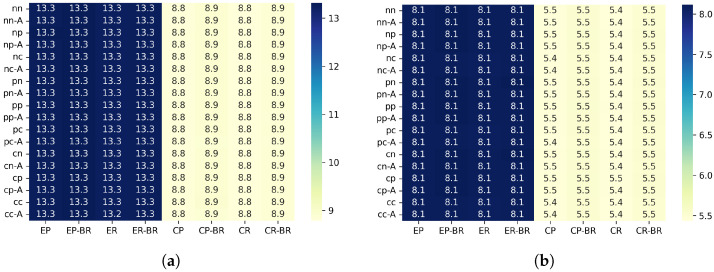
End-to-End Latency (s) BI5 and BI3 configurations. (**a**) BI5. (**b**) BI3.

Figure 11 show the end-to-end delay for each configuration.

In this evaluation, no significant differences in end-to-end delay were observed across scenarios or configurations. In this simulation, MAC-layer retransmissions are disabled, so when a transmission failure occurs, the packet is immediately dropped and is not counted as part of the delay. Because of this design, all successfully delivered packets are those that succeeded in a single attempt, making it unlikely for delay differences to appear between configurations.

In addition, the node placement and traffic load used in this study were relatively stable, and the propagation paths of successful packets did not vary significantly. This also contributes to the uniformity of delay results across configurations.

On the other hand, if retransmissions were enabled, differences in contention and channel utilization would affect the number of retries, and the delay characteristics of each configuration would likely become more apparent. Evaluations under higher load, higher density, or with MAC-layer retransmissions would allow for a more detailed understanding of the delay behavior of the proposed framework.

### 6.5. Discussion and Design Guidelines

Based on the simulation results obtained in this study, we summarize several design guidelines for RIT-based MAC protocols as follows.

- Beacon randomization (“-BR”) should be applied carefully in low-load environments.
  Under the evaluation conditions of this study, BR increased the average awake ratio and led to PDR degradation in the Edge scenario. In low-load, many-to-one network configurations, BR cannot provide its intended desynchronization effect, and the fluctuation of the beacon interval instead increases temporary waiting time. Therefore, in low-load networks, the use of BR should be carefully judged depending on the node placement.
- CSMA/CA is effective for data transmission, and Pre-CS may be useful for beacon transmission.
  For data transmission, CSMA/CA helps mitigate sender contention and provides a stable improvement. For beacon transmission, differences in channel access mechanisms did not significantly affect PDR or delay under the low-load and low-density conditions of this study. However, in real deployments, external noise and interference from other users may cause beacon collisions. In such cases, Pre-CS, which has smaller transmission delay, may be more advantageous. The choice should consider both the operating environment and the balance with energy consumption.
- Beacon ACK may degrade performance but significantly reduces energy consumption.
  Beacon ACK can reduce PDR because the ACK response increases channel occupancy and may introduce additional contention. However, in the Edge scenario, where traffic concentration near the parent is relatively small, this negative impact is limited, and the reduction in energy consumption becomes more dominant. In contrast, in dense topologies such as the Center scenario, the risk of losing the channel before ACK transmission is higher due to interference or hidden nodes. This may lead to prolonged CSMA/CA delay or transmission failures. Therefore, the use of beacon ACK should be carefully decided based on the topology and interference environment.

## 7. Conclusions

In this study, we aimed to provide a unified way to compare improvement mechanisms in receiver-initiated MAC protocols. To achieve this, we implemented an IEEE 802.15.4e RIT-compliant MAC layer in ns-3 and developed an evaluation platform that allows flexible configuration of multiple enhancement modules. Using this platform, we conducted comprehensive simulation comparisons by combining different application traffic patterns, node placements, beacon intervals, and the presence or absence of each improvement mechanism.

As a result, we found that the PDR and the average awake ratio change significantly depending on the application characteristics and network structure. In particular, in low-load environments, the previously proposed beacon randomization (BR) did not show the expected effect of avoiding unwanted synchronization, and sometimes degraded performance due to increased waiting time caused by variations in the beacon interval. On the other hand, CSMA/CA for data transmission showed stable effectiveness in all configurations, confirming that it works as a fundamental contention-avoidance method in receiver-initiated MAC protocols. We also confirmed that the effectiveness of the beacon ACK depends on the topology and interference conditions, and that its use should be carefully decided based on the environment.

In addition, many previous studies on receiver-initiated MAC protocols have focused on proposing individual improvement mechanisms or showing their effectiveness in specific scenarios. However, a unified framework for fairly comparing multiple mechanisms under the same conditions and organizing their applicable range and performance limits has not been well established. The evaluation platform developed in this study enables quantitative analysis of these unexplored aspects, and allows us to clarify the effective conditions and applicability boundaries of each improvement mechanism.

On the other hand, several issues remain for future work. First, the gap between simulation and real devices needs to be reduced. The clock-drift model and radio switching delay used in this study are simplified, so a more realistic model that reflects the asynchronous behavior observed in real hardware is required. Second, since our evaluation mainly focused on low-load environments and limited parameter settings, it is necessary to conduct experiments under a wider variety of topologies, load conditions, and traffic patterns to understand the applicability and performance boundaries of each improvement mechanism. Third, the current logging system can record only a limited set of metrics. To analyze internal protocol behavior in detail, such as the causes of transmission failures, the breakdown of waiting time, and where contention occurs, more advanced data collection functions are needed. Fourth, due to the multi-layered combination of application settings, topologies, protocol parameters, and improvement mechanisms, the design space becomes extremely large. Developing guidelines for efficient evaluation, such as representative scenario extraction or automated exploration methods, is an important future challenge.

In summary, the RIT-based evaluation platform developed in this study is a useful foundation for comparing improvement mechanisms of receiver-initiated MAC protocols under transparent and consistent conditions. We expect that this platform will contribute to understanding the performance boundaries under different operating conditions, and will support future research on protocol design and improvement.

## Figures and Tables

**Table 1:** Input Parameter.

Parameter	Values
Simulation Time	1 days
User Data Generation Interval	300 s, 180∼600 s
User Data Payload Length	8 bytes
RIT MAC Period Time	1 s (Sink Node), 3 s, 5 s
RIT Tx Wait Duration	3 s, 5 s
RIT Data Wait Duration	2 ms (BeaconAck), 5 ms
Clock Drift Skew	±250 ppm
Propagation Loss Model	LogDistancePropagationLossModel
Propagation Delay Model	ConstantSpeedPropagationDelayModel

**Table 2:** Evaluation Metrics.

Metric	Description
PDR (Packet Delivery Ratio)	Percentage of successfully delivered packets (%)
Wake-Up Ratio	Percentage of time during which each node is awake (%)
End-to-End Latency	Time from packet generation to successful reception (s)

**Table 3:** Scenario Types and Beacon Randomization Settings Used for the Horizontal Axis.

Label	Density	Application	Beacon Randomization
EP	Edge	Periodic	OFF
EP-BR	Edge	Periodic	ON
ER	Edge	Randomization	OFF
ER-BR	Edge	Randomization	ON
CP	Center	Periodic	OFF
CP-BR	Center	Periodic	ON
CR	Center	Randomization	OFF
CR-BR	Center	Randomization	ON

**Table 4:** CSMA Configuration and Beacon ACK Settings Used for the Vertical Axis.

Label	Data	Beacon	Beacon ACK
nn	None	None	OFF
nn-A	None	None	ON
np	None	Pre-CS	OFF
np-A	None	Pre-CS	ON
nc	None	CSMA/CA	OFF
nc-A	None	CSMA/CA	ON
pn	Pre-CS	None	OFF
pn-A	Pre-CS	None	ON
pp	Pre-CS	Pre-CS	OFF
pp-A	Pre-CS	Pre-CS	ON
pc	Pre-CS	CSMA/CA	OFF
pc-A	Pre-CS	CSMA/CA	ON
cn	CSMA/CA	None	OFF
cn-A	CSMA/CA	None	ON
cp	CSMA/CA	Pre-CS	OFF
cp-A	CSMA/CA	Pre-CS	ON
cc	CSMA/CA	CSMA/CA	OFF
cc-A	CSMA/CA	CSMA/CA	ON

**Table 5:** Theoretical maximum aggregate throughput for each scenario.

Scenario	Number of Nodes	Maximum Throughput [bps]
EP	45	9.60
ER	45	7.38
CP	48	10.24
CR	48	7.87

## Data Availability

The implementation for an open-source network simulator (ns-3), including newly added and modified components, as well as the analysis scripts used in this study, are publicly available via a GitHub repository.
